# A Novel Baseline Removal Paradigm for Subject-Independent Features in Emotion Classification Using EEG

**DOI:** 10.3390/bioengineering10010054

**Published:** 2023-01-01

**Authors:** Md. Zaved Iqubal Ahmed, Nidul Sinha, Ebrahim Ghaderpour, Souvik Phadikar, Rajdeep Ghosh

**Affiliations:** 1 Department of Computer Science & Engineering, National Institute of Technology, Silchar 788010, India; 2 Department of Electrical Engineering, National Institute of Technology, Silchar 788010, India; 3 Department of Earth Sciences and CERI Research Center, Sapienza University of Rome, Piazzale Aldo Moro, 5, 00185 Rome, Italy; 4 Neurology Department, University of Wisconsin-Madison, Madison, WI 53705, USA; 5 School of Computing Science and Engineering, VIT Bhopal University, Bhopal 466114, India

**Keywords:** EEG, inverse filtering, baseline removal, emotion classification

## Abstract

Emotion plays a vital role in understanding the affective state of mind of an individual. In recent years, emotion classification using electroencephalogram (EEG) has emerged as a key element of affective computing. Many researchers have prepared datasets, such as DEAP and SEED, containing EEG signals captured by the elicitation of emotion using audio–visual stimuli, and many studies have been conducted to classify emotions using these datasets. However, baseline power removal is still considered one of the trivial aspects of preprocessing in feature extraction. The most common technique that prevails is subtracting the baseline power from the trial EEG power. In this paper, a novel method called InvBase method is proposed for removing baseline power before extracting features that remain invariant irrespective of the subject. The features extracted from the baseline removed EEG data are then used for classification of two classes of emotion, i.e., valence and arousal. The proposed scheme is compared with subtractive and no-baseline-correction methods. In terms of classification accuracy, it outperforms the existing state-of-art methods in both valence and arousal classification. The InvBase method plus multilayer perceptron shows an improvement of 29% over the no-baseline-correction method and 15% over the subtractive method.

## 1. Introduction

Emotions have the potential to improve the effectiveness of human interaction, whether it is human-to-human or human-to-machine. Emotions have a profound impact on human cognition, including logical decision making, perception, human interaction, and intelligence [1,2]. However, modeling human emotion based on the mechanism behind the emotional function of the brain is a challenging task [3]. In the last decade, human–machine interaction (HMI) has received much attention. However, while interacting with a machine, emotional communication is almost nonexistent compared with that between humans. As we are strongly associated with machines (especially computers), it has become essential to involve emotion in HMI. According to Rani et al., HMI may be more intuitive, smoother, and effective in creating a new approach in the affective, cognitive, and developmental systems if machines can grasp a person’s affective state [4]. At the core of such systems lies the problem of emotion recognition, which is to identify human emotional states from their behavioral and physiological signals [5]. These human emotions can be vital information for HMI, biomedical research, and others. EEG-based emotion recognition can help improve patient treatment, especially those with expression problems and depression, as it will help the doctor with identifying the real emotional state of the patients [6].

Emotion classification, in general, is the process of classifying an individual’s emotional state. There are several ways to record brain activity, but EEG has gained enormous popularity because it is noninvasive, portable, affordable, and applicable to practically all settings [7]. However, EEG signals are very complex, and human emotions are very ambiguous, making the high-accuracy classification of emotion a challenging task. There are various preprocessing techniques for EEG data, such as downsampling and denoising. Some of the standard denoising techniques in EEG are bandpass filtering for removing external noise, eye-blink artifact removal, and baseline removal. A baseline is the EEG signal generated from the brain during the relaxed state of an individual. The baseline blurs the intended EEG signal corresponding to a stimulus; thus, baseline removal is an essential preprocessing step for denoising EEG signals. The main motivation in performing baseline removal is to refine the EEG signals before extracting features. The baseline-removed EEG signals does not carry the subject-specific noise, thus resulting in subject-independent features.

This paper proposes a novel method called the InvBase method for extracting subject-independent features for emotion classification. The method employs the concept of inverse filtering for baseline removal. However, inverse filtering is a common method in stationary signals such as images. Its application for baseline removal in nonstationary signals, such as EEG, is considered a significant contribution of this study. This method exploits the baseline recording of the benchmark DEAP dataset captured during the relaxed state of an individual, where DEAP stands for database for emotion analysis using physiological signals [8]. The power spectrum of the baseline is used to eliminate the excess power in the trial EEG power captured during an emotional event. The proposed method for baseline removal utilizes the idea of inverse filtering, which is commonly used in denoising blurred images [9,10]. In the proposed method for baseline removal, an EEG signal is first split into fixed-size time slots. The time-domain EEG signals corresponding to each time slot are then converted to frequency-domain signals. The frequency-domain signals are baseline removed using inverse filtering. These baseline signals are grouped in nonoverlapping time windows and averaged. For each window, the trend and harmonic are simultaneously fit to segments in order to estimate the power of the residual segments [11]. Necessarily, the window size is greater than the slot size. After that, the frequency spectrum in each window is subdivided into four frequency sub-bands for each channel, and statistical features, such as mean and variance, are extracted as features. These features are considered to be subject-independent as the individual’s EEG data are filtered with the removal of baseline data of the individual, thus retaining only the EEG characteristics corresponding to the particular emotion. Following that, these features are used to train three different classifiers: k-nearest neighbour (kNN), support vector machine (SVM), and multilayer perceptron (MLP). In this study, two classification problems were taken into consideration, (1) high arousal vs. low arousal and (2) high valence vs. low valence.

The baseline removal technique, which subtracts the frequency spectrum of the baseline from the frequency spectrum of the EEG signal, was also implemented in this study and is termed the subtractive method. The InvBase method was compared with the subtractive method and the no-baseline-correction (NBC) method. The NBC method does not remove the baseline from the EEG data. Various validation analyses were performed on all the methods.

The novel InvBase method, used to extract subject-independent features, can be further implemented for other EEG-based classification problems, such as cognitive load estimation and motor imagery. However, the technique was employed in this study to remove the baseline from EEG data in order to classify emotions. It is evident after observing the DEAP dataset [8] that EEG signals vary from subject to subject for the same elicited emotion. Furthermore, performing feature extraction in such data generates subject-dependent features that hamper the classification accuracy. Emotion-related EEG features are highly subject-dependent due to the presence of a baseline. In order to obtain subject-independent features for EEG-based emotion classification, the InvBase method shows considerable potential.

The remainder of the paper is organized as follows: In Section 2, a detailed review of the studies in the field of emotion classification is presented. The background of the current study is elaborated in Section 3. In Section 4, the proposed InvBase method for baseline removal and feature extraction process is discussed in detail, and the classification problem is also elaborated. The experimental results are provided in Section 5. Finally, the discussion and conclusions are presented in Section 6 and Section 7, respectively.

## 2. Literature Survey

In this section, we discuss the different aspects of EEG-based emotion classification research. EEG-based emotion classification requires the following actions: emotion elicitation and signal acquisition, preprocessing, feature extraction, and classification.

The two major techniques used for emotion elicitation are using external stimuli, such as audio–visual [8,12,13] or memory recall [14]. For signal acquisition, BiosemiActive Two, Emotiv wireless headset, EEG module from Neuroscan Inc., and g.MOBIlab are the most used devices [15]. The preprocessing step comprises downsampling, eye-blink artifact removal [16], electromyogram artifact removal [17], baseline removal [8,18,19], bandpass filtering for noise removal, and others. Various researchers have also used wavelet-transform-based denoising techniques for EEG signals [20].

After preprocessing the EEG signals, the next important step is feature extraction. Features are frequently derived from the delta, theta, alpha, beta, and gamma frequency regions for emotion classification. The following feature extraction techniques are usually used for emotion classification: asymmetry measure (ASM) [21], power spectral density (PSD) [13], differential entropy (DE) [21], wavelet transform (WT) [22], higher-order crossings (HOC) [23], common spatial patterns (CSP) [17], asymmetry index (AI) [24], and AsMap [25]. Furthermore, in the least-squares wavelet analysis, features are extracted from time series data without the need for editing or preprocessing of the original series [26].

In this study, frequency sub-bands features were extracted as they are the most widely used features in EEG research. Emotion classification is the final step in which the extracted features are used to train a classifier. Classification tools, such as SVM [13,17,21,27,28], linear discriminant analysis [29,30], quadratic discriminant analysis [23], k-NN [21,22,23,31], naïve Bayes [30], feed-forward neural network [32], deep belief network [1], multilayer perceptron neural network (MLPNN) [22], convolution neural network (CNN), and recurrent neural network (RNN) [19] are frequently used in EEG-based emotion classification. Fraiwan et al. in [33] proposed an ANN-based machine learning model for classifying enjoyment levels of individuals. Their model uses multiscale entropy (MSE) to calculate features, such as mean MSE, slope of the MSE, and complexity index for emotion classification. However, these researchers did not consider any baseline removal technique for eliminating unwanted noise in the EEG signals.

Later in this section, the preprocessing technique that involves baseline removal before extracting features for emotion classification is discussed. Fewer studies have been reported in this area, as baseline removal is considered trivial preprocessing. A dataset, namely DEAP, was created by Koelstra et al., which contains EEG and physiological information from subjects exposed to audio–visual stimuli [8]. In their study, they recorded a 5 s baseline EEG in a relaxed state, and a 60 s music video was played during which EEG data were recorded. The baseline frequency power was subtracted from each trial’s frequency power. The frequency power was calculated between 3 and 47 Hz. The subtractive method in this study calculates the change in power compared with the prestimulus time. Theta (3–7 Hz), alpha (8–13 Hz), beta (14–29 Hz), and gamma (30–47 Hz) frequency bands were summed to provide these variations in power, which were then deployed as features to train a Gaussian naïve Bayes classifier for low/high arousal, valence, or liking. Lastly, the accuracies of the EEG-based classification for arousal, valence, and liking were 62.0%, 57.6%, and 55.4%, respectively.

Xu et al. [18] suggested a fundamental method for deriving emotional traits from EEG. The strategy’s core element is to rectify the emotional data by filtering the baseline data. To verify their method, they employed the DEAP dataset. The baseline data are first converted into a frequency spectrum, and correlation coefficients are calculated for the baselines of a subject. Highly correlated baselines are retained, and those weakly correlated are replaced by the mean of the highly correlated baselines. After that, each trial’s power spectral density (PSD) is corrected based on the PSD of the original high-correlation baseline and the new baseline. However, Xu et al. did not directly mention the baseline removal method. In order to determine the PSD’s mean, maximum, minimum, standard deviation, skewness, kurtosis, and fractal dimension, the frequency spectrum was divided into five segments: theta (4–7 Hz), alpha (8–12 Hz), lower beta (13–21 Hz), upper beta (22–30 Hz), and gamma (31–45 Hz). From each channel, 35 characteristics, or 1120 features in total, were obtained by the aforementioned methods. The SVM with a radial basis kernel function was then used as the classifier. Additionally, the PSD features were used to train a CNN. The arousal classification accuracies obtained using the baseline strategy on SVM and CNN were 79.54% and 77.69%, respectively. Furthermore, using the baseline strategy, the valence classification accuracies obtained on SVM and CNN were 75.62% and 81.14%, respectively.

Yang et al. [19] put forward a preprocessing method based on baseline signals. In their study, they built a hybrid network that combines CNN and RNN to classify emotions. In addition to the preprocessing conducted in the DEAP dataset, they further processed the EEG signal. From the baseline signal, the baseMean is calculated by segmenting the signal into N segments of L length for each C channel. Each segment is a C × L matrix. All the C × L matrices are added lengthwise, and mean values are calculated, which is termed as baseMean. The baseMean is then subtracted from the raw EEG signal segmented likewise. The 1D EEG vector is then transformed into a 2D vector or an EEG frame in a subsequent step that preserves spatial information between many neighboring channels. After that, each data frame is normalized across the nonzero element using Z-score normalization. The 2D EEG frames are fed in parallel to CNN and RNN to obtain the spatial feature vector (SFV) and temporal feature vector (TFV), respectively. The SFV and TFV are concatenated and fed into a SoftMax function to classify valence and arousal. The highest classification accuracies achieved for valence and arousal were 90.80% and 91.03%, respectively.

## 3. Background

In this section, the DEAP dataset is discussed again as it was used as the benchmark dataset in this study to evaluate the proposed method. Additionally, the inverse filtering technique is discussed in detail as it is exploited in the proposed method to eliminate baseline power.

### 3.1. DEAP Dataset

A multimodal dataset termed DEAP that includes EEG and physiological signals was created by Koelstra et al. [8]. The dataset, which has a balanced women-to-men ratio, was created from the recordings of 32 individuals, who ranged in age from 19 to 37 years. An emotional response was obtained from each participant using 40 videos. Out of 120 music videos collected from the website last.fm using effective tags, 40 videos were manually chosen. A web-based subjective emotion assessment interface was used during the video selection process. All of the videos contained music videos and were one minute long. A sampling rate of 512 Hz was used while capturing the EEG using 32 electrodes (placed according to the international 10–20 system). A total of 13 peripheral physiological data, including the electromyograms of the zygomaticus and trapezius muscles, the respiration amplitude, the skin temperature, GSR, and the electrooculogram (EOG), were also recorded. In the method, the videos were trialed with each participant:

- First, the current trial number was displayed for 2 s.
- A 5 s baseline recording was taken in a relaxed state.
- Then, a 1 min music video was displayed.
- Finally, the participants gave their ratings on a discrete nine-point scale for valence, arousal, and dominance. To rate their liking, thumbs down/thumbs up symbols were used.

The DEAP dataset includes the eye blink artifact free, preprocessed EEG recordings that were further downsampled to 128 Hz. Additionally, a 4.0–45.0 Hz bandpass filter was used. The data were split into 60 s trials and a 3 s pretrial baseline. Furthermore, the participant ratings for valence, arousal, and dominance are provided along with the dataset.

### 3.2. Inverse Filtering

**Figure 1:**
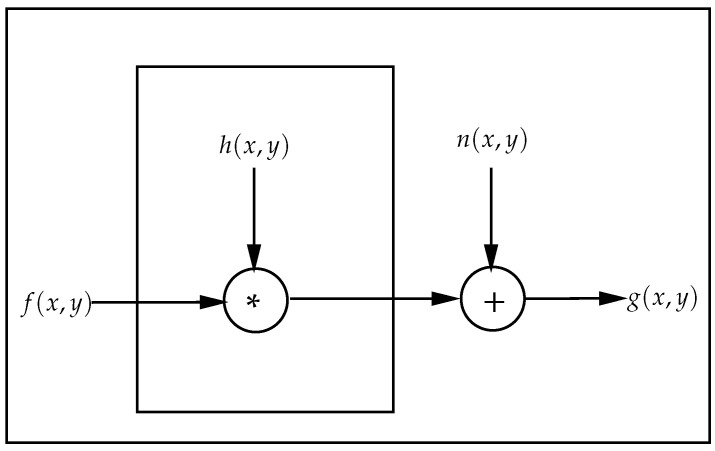
Modeling the distortion/degradation of a signal.

In engineering, inverse filtering is a frequently used method for reconstructing the original scene from damaged observations [34]. Using inverse filtering, the estimated signal, $\hat{f}(x,y$) is obtained from an observed signal g(x,y) so that $\hat{f}\left(x,y\right)\approx f\left(x,y\right)$. Here, f(x,y) is the original undistorted signal. The degradation/distortion of the signal f(x,y) is illustrated in Figure 1, where

(1) g(x,y)=f(x,y)∗h(x,y)+n(x,y)

Here, h(x,y) is the distortion/degradation function, and n(x,y) is the noise introduced to the signal due to external factors (in ideal situation n(x,y) can be considered to be zero). The point-spread function, $h^{{}^{\prime }}\left(x,y\right)$ is an inverse filter that is the inverse of the degradation function, h(x,y), in the sense that

(2) $$h\left(x,y\right)*h^{{}^{\prime }}\left(x,y\right)=\delta \left(x,y\right)$$

According to the convolution theorem, under suitable conditions, the Fourier transform of a convolution of two signals is the point-wise product of their Fourier transforms. The spectral counterpart of Equation (2) can be given as

(3) $$\begin{array}{cc} H\left(u,v\right)H^{{}^{\prime }}\left(u,v\right) & =1\\ \Rightarrow H^{{}^{\prime }}\left(u,v\right) & ={\left(H\left(u,v\right)\right)}^{-1}=\frac{1}{H(u,v)} \end{array}$$

In inverse filtering, the spectral counterpart of g(x,y), given as G(u,v) when multiplied with $H^{{}^{\prime }}\left(u,v\right)$, results in $\hat{F}\left(u,v\right)$ (spectral counterpart of $\hat{f}\left(x,y\right)$). Mathematically,

(4) $$\hat{F}\left(u,v\right)=H^{{}^{\prime }}\left(u,v\right)G\left(u,v\right)$$

In Equation (4), $\hat{F}\left(u,v\right)$, the spectral representation of $\hat{f}\left(x,y\right)$, is obtained. The inverse filter has the advantage of only requiring prior knowledge of the blur point-spread function, enabling flawless restoration in the absence of noise.

## 4. Proposed Method

In this section, we discuss the proposed InvBase method of baseline removal from EEG recordings. Different subjects have different baseline powers, which leads to the generation of different features for different subjects but for the same stimulus. This, in turn, leads to poor classification accuracy in EEG-based classification of emotions. Therefore, removing the baseline power from each subject can lead to the generation of subject-independent features. The steps involved with InvBase method are given below:

- Baseline removal;
- Windowing;
- Feature extraction.

### 4.1. Baseline Removal

As EEG signals are recorded from different individuals, the features extracted from nonbaseline removed EEG signals can lead to the generation of features that are specific to the individual. Thus, the features remain subject-dependent. We hypothesized that by performing baseline removal, subject-independent features can be obtained. We used the benchmark DEAP dataset to explore the baseline removal process. In the dataset, each EEG recording comprises two parts: 60 s EEG data recorded during stimuli presentation and 5 s EEG data recorded when in a relaxed state. The relaxed-state EEG is considered the baseline of the subject. Although the authors of the DEAP dataset mentioned a baseline recording of 5 s in [8], only 3 s baseline data were included in the publicly available dataset. Therefore, the proposed method considered the 3 s baseline EEG data during the experimentation.

The proposed baseline removal method from EEG signals, termed InvBase, is a process of point-wise division of the frequency power of the contaminated EEG signal over the entire spectrum by the frequency power of the relaxed-state EEG signal. EEG signals recorded in the relaxed state do not contain emotion information; they should contain only baseline information. In the signal degradation model depicted in Figure 1, if f(x,y) is considered as the relaxed/resting-state EEG signal, then g(x,y) should be δ(x,y), which implies that the output signal has no emotion information. With the assumption of zero noise and no emotion information in the output EEG signal, we can rewrite Equation (1) with n(x,y)=0 and g(x,y)=δ(x,y) as

(5) f(x,y)∗h(x,y)=δ(x,y)

From Equations (3) and (5), we can further be conclude that $f\left(x,y\right)=h^{{}^{\prime }}\left(x,y\right)$. Therefore, the frequency power of resting-state EEG signals can be considered as the $H^{{}^{\prime }}$ of inverse filtering. The proposed InvBase method uses inverse filtering for baseline power removal from each channel. The 60 s EEG recording of a channel, x(t), is split into *N* equal-sized nonoverlapping time slots, $x(t_{i})$, such that $x\left(t\right)=\left[x\left(t_{1}\right),x\left(t_{2}\right),x\left(t_{3}\right),\cdots ,x\left(t_{N}\right)\right]$.

The frequency spectrum corresponding to each slot, $x(t_{i})$, is obtained using fast Fourier transform (FFT). Let ${\mathrm{FFT}}_{i}\left(v\right)$ be the frequency spectrum for the *i*th time slot. The resting-state EEG signal containing baseline data for a particular channel are considered as $h^{{}^{\prime }}$ in Equation (2). Furthermore, FFT is applied on $h^{{}^{\prime }}$ to obtain the baseline frequency spectrum, ${\mathrm{FFT}}_{base}\left(v\right)$, which is analogous to $H^{{}^{\prime }}$. Then ,using the concept of inverse filtering, the baseline-free frequency spectrum is obtained by dividing the frequency spectrum of each slot by the frequency spectrum of the baseline signal. Here, the external noise is assumed to be zero, as preprocessing was performed during the construction of the DEAP dataset. Equation (6) depicts the baseline-eliminated spectrum achieved through inverse filtering.

(6) $${\mathrm{FFT}}^{baseRem}\left(v\right)=\frac{{\mathrm{FFT}}_{i}\left(v\right)}{{\mathrm{FFT}}_{base}\left(v\right)}$$

**Figure 2:**
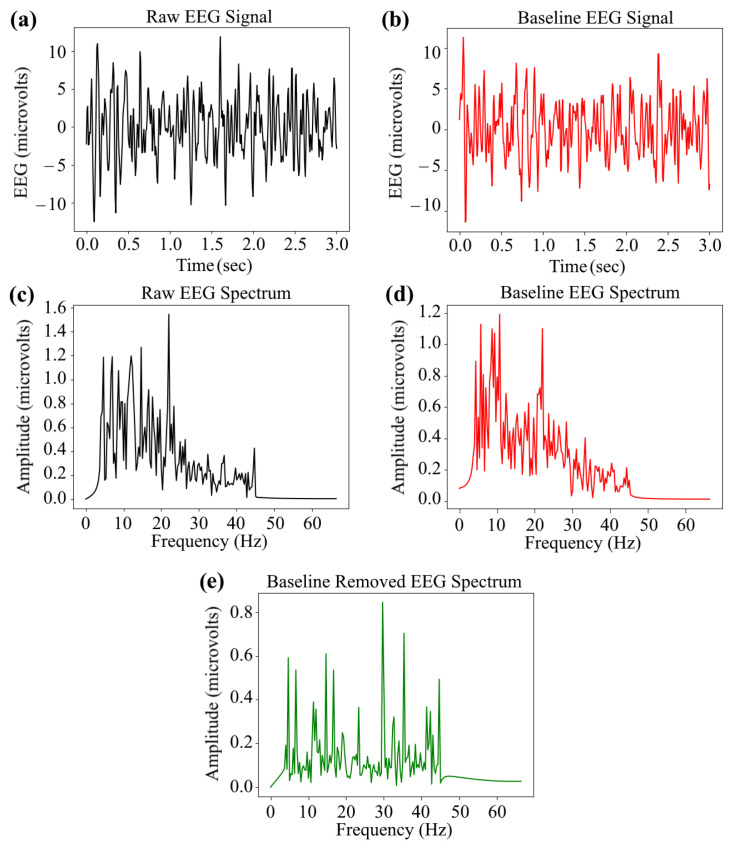
An example of baseline EEG removal results: (**a**) raw EEG signal in a 3 s time slot, (**b**) baseline EEG signal captured for 3 s, (**c**) frequency spectrum of the raw EEG signal, (**d**) frequency spectrum of the baseline EEG signal, and (**e**) the baseline removal in a 3 s time slot from a channel using the InvBase method.

The InvBase method of baseline removal from a 3 s EEG time slot is depicted in Figure 2. The time-domain EEG signal in a 3 s time slot on a particular channel in Figure 2a and its corresponding baseline signal on that channel in Figure 2b are transformed into their respective frequency domain using FFT. The frequency spectra of the raw EEG signal and baseline signal are shown in Figure 2c,d, respectively. The frequency spectra of both signals are fed into the baseline removal process. The output of the process shown in Figure 2e is the baseline-removed frequency spectrum for the particular time slot.

### 4.2. Windowing

**Figure 3:**
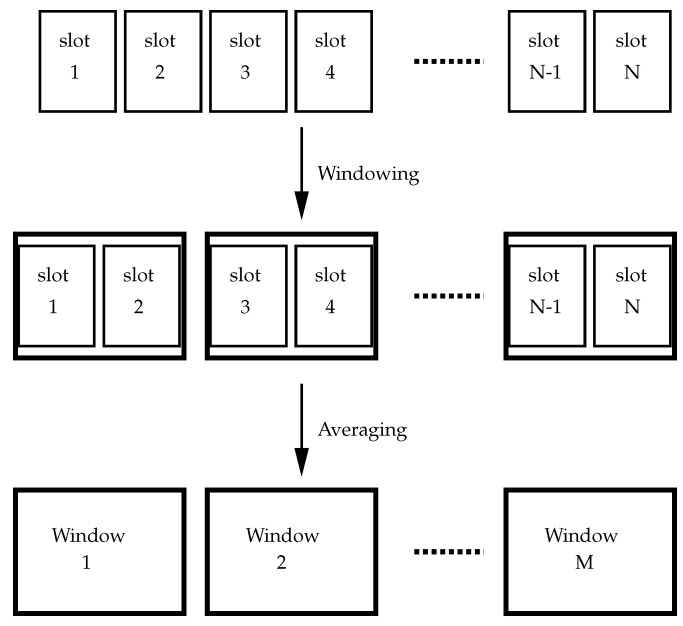
The windowing process.

After baseline removal, the next action is to group the consecutive baseline-removed frequency spectra from each slot in fixed-size windows and average the frequency spectrum. Previous studies have demonstrated that emotions have a short-term memory, which means that they last for sometime until the next emotional stimulation [32,35]. Most researchers employed 1 to 4 s EEG signals to determine emotional states because short-term EEG signals are typically thought to be stable [35]. In this step, we mainly concentrated on temporal memory features connected to emotions and investigated the effects of various time windows on EEG characteristics. The characteristics of emotion EEG in consecutive time slots are considered to have similar behavior; thus, the slots are grouped into fixed windows. This operation of grouping slots and averaging generalizes the frequency spectrum over a period of time equal to the window size. Figure 3 demonstrates the windowing process in the InvBase method. The averaged data obtained in each window for all the channels are considered a data point for the same class to which the original EEG dataset belongs.

### 4.3. Feature Extraction

The baseline-removed frequency spectrum in a time window is subdivided into four frequency sub-bands. Frequencies from 3 to 7 Hz correspond to the theta band, 8 to 13 Hz to the alpha band, 14 to 29 Hz to the beta band, and from 30 to 47 Hz to the gamma band. Two statistical features, mean and variance, are calculated on each frequency band in each time window to obtain the spectral features. A total of 8 features are extracted from each time window. The extraction process is performed on all the 32 channels in the same time window, and the features are concatenated to form the feature vector containing 256 features. Each feature vector contains the mean and variance on each sub-band corresponding to each channel.

Two dimensions of emotion, valence and arousal, from the dimensional model, were used for emotion classification. Valence relates to feelings that range from unpleasant or depressed to joyful. Arousal also relates to feelings that range from boredom to enthusiasm. Each EEG recording in the DEAP dataset has a corresponding subjective rating for valence and arousal. The resulting spectral features are divided into two binary classes for both valence and arousal based on the distribution of the participants’ subjective assessments [8]. Ratings from 1 to 5.5 in valence are classified as low-valence (LV) values, whereas valence ratings from 5.5 to 9 are classified as high-valence (HV) ratings. Similarly, the participant’s ratings between 1 and 5.5 in arousal are classified as low-arousal (LA) ratings, whereas those between 5.5 and 9 in arousal are classified as high-arousal (HA). To give an indication of the chance level of the classifiers, the sample size of each class is reported in Table 1. The spectral features were then used to train three different classifiers, SVM, kNN, and MLP, and their classification accuracies and F1 scores were calculated.

## 5. Results

In this section, the setup used in the experiment is discussed. Furthermore, the results obtained in terms of classification accuracy and F1 score are presented. The InvBase method was compared with the subtractive and NBC methods in two stages for both arousal and valence classification.

### 5.1. Experimental Setup

An Acer desktop computer with an Intel Core i3 seventh-generation processor with 4 GB RAM was used for the experiment. Scientific computing was carried out using Anaconda 3, which is an open-source distribution of the Python and R programming languages. Some of the crucial Python modules used for data handling during the experiment were Numpy, Pandas, and Scikit-Learn. The EEG recordings of the DEAP dataset, except for the physiological signals of the 32 subjects who saw the 40 videos, were used in the experiment. Two other methods, the subtractive and NBC methods, were also implemented for comparison of their performance with that of the InvBase method. In the subtractive method, the frequency spectrum ofthe baseline is subtracted from the frequency spectrum of each slot [8]. In the NBC method, the features are directly extracted from the frequency spectrum of each slot without baseline removal. The InvBase method was compared with both methods. Slot size and window size are two important parameters that were evaluated. Slot size and window size were varied to explore the classification accuracy of all the methods. Three classifiers, MLP, SVM, and kNN, were separately trained on the InvBase, subtractive, and without-baseline-correction methods. Throughout the experiment, the MLP contained two hidden layers: one with 64 and the other with 32 neurons. The radial basis function served as the kernel for the SVM in this experiment. The algorithm used in kNN was kd-tree, and the number of neighbors was set to five. Each model was validated using 10-fold cross-validation. The arousal and valence classifications were separately performed on the different models. Classification accuracy and F1 score were used as performance measure. The classification accuracy was determined by dividing the number of correct predictions by the total number of predictions. The harmonic mean of recall and precision was used to calculate the F1 score (e.g., see [36] for their mathematical definitions).

### 5.2. Classification Accuracy with Varying Slot Size

**Figure 4:**
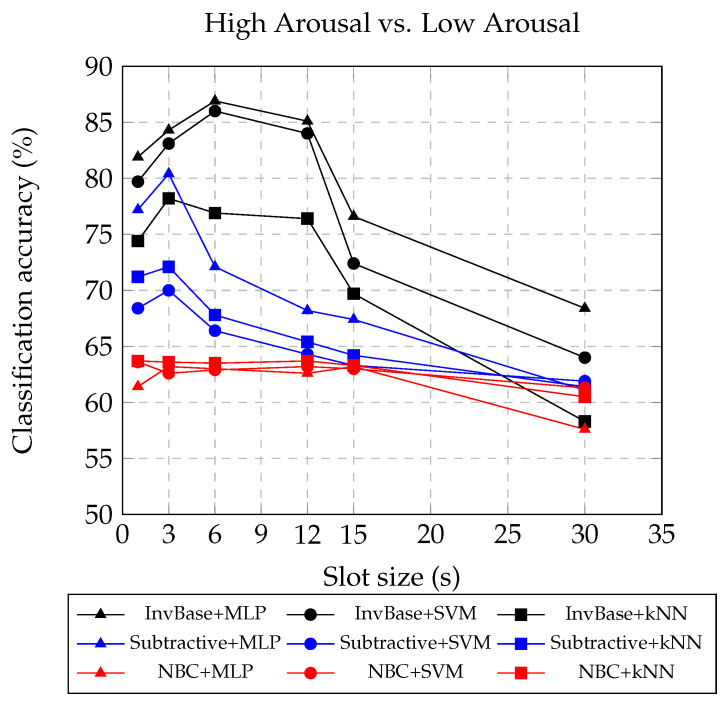
Arousal classification accuracies obtained using different slot sizes in InvBase, subtractive, and NBC methods.

The arousal classification accuracies and F1 scores of the InvBase, subtractive, and NBC methods with SVM, kNN, and MLP as classifiers for slot sizes 1, 3, 6, 12, 15, and 30 s are presented in Table 2, Table 3 and Table 4, respectively. The window size was fixed at 12 s for 1 s, 3 s, 6 s, and 12 s slot sizes. For slot sizes above 12 s, the window size was same as the slot size. Therefore, the window sizes for slot sizes 15 s and 30 s were set to 15 s and 30 s, respectively. From Figure 4, it is observed that the 6 s slot had the highest arousal classification accuracy with the InvBase method using MLP. The arousal classification accuracies of the InvBase, subtractive, and NBC methods using the MLP classifier on the 6 s slot size were 86.9%, 72.1%, and 63%, respectively. The InvBase method+MLP showed an improvement of 24% over the NBC method and 15% over the subtractive method. The arousal classification accuracies of the InvBase, subtractive, and NBC methods using the SVM classifier on the 6 s slot size were 86%, 66.4%, and 62.9%, respectively. The InvBase method+SVM showed an improvement of 23% over the NBC method and 20% over the subtractive method. The arousal classification accuracies of the InvBase, subtractive, and NBC methods using the kNN classifier on the 6 s slot size were 76.9%, 67.8%, and 63.5%, respectively. The InvBase method+kNN showed an improvement of 9% over the NBC method and 13% over the subtractive method. For all the classifiers, the InvBase method outperformed the other methods. From Figure 4, it is also evident that the slot size had an impact on arousal classification accuracy. As the slot size increased beyond 12 s, the arousal classification accuracy deteriorated because the increase in slot size impacted the variability in the features in the frequency domain. It is also observed that the InvBase method produced superior arousal classification accuracy with MLP and SVM than with kNN. Additionally, the F1 score for the InvBase method+MLP with the 6 s slot was the highest and validated the arousal classification accuracy.

**Figure 5:**
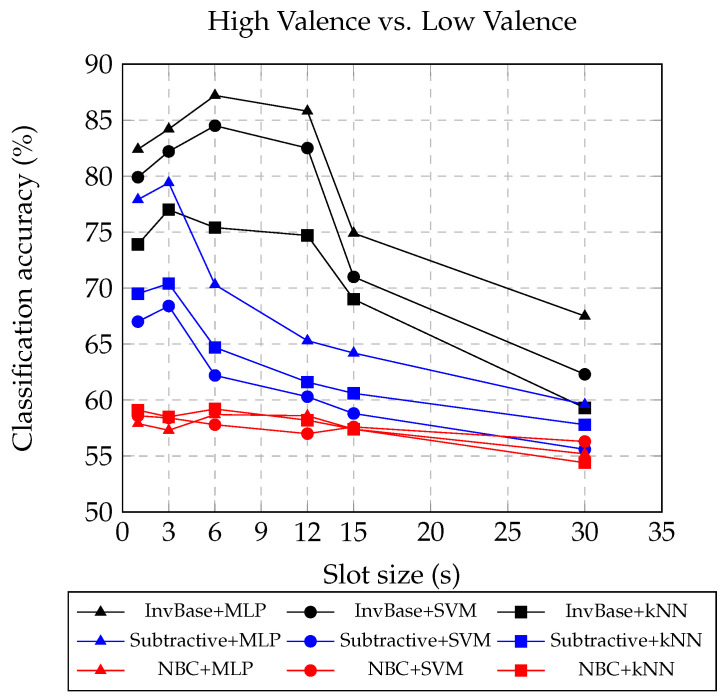
Valence classification accuracies obtained using different slot sizes in InvBase, subtractive, and NBC methods.

Table 5, Table 6 and Table 7, respectively, present the valence classification accuracies of MLP, SVM, and kNN with the InvBase, subtractive, and NBC methods for various slot sizes (keeping the window size fixed for slot size up to 12 s). For slot sizes above 12 s, the window size was same as the slot size. Therefore, the window sizes for slot sizes 15 s and 30 s were set to 15 s and 30 s, respectively. Figure 5 presents the valence classification accuracies obtained using different slot sizes in the InvBase, subtractive, and NBC methods. The figure captures the classification accuracies obtained by using the MLP, SVM, and kNN classifiers. It shows results similar to the arousal classification. The highest valence classification accuracy was 87.2% with the InvBase method+MLP with a 6 s slot. With a 6 s slot, the subtractive method+MLP had 70.3% accuracy and NBC+MLP had 58.7% classification accuracy. The improvement in valence classification accuracy using the InvBase method was 17% and 29% over the subtractive and NBC methods, respectively. The valence and arousal classification accuracies with the InvBase method for a 6 s slot were close. However, the valence classification accuracy for NBC dropped by 4.3% compared with the arousal classification accuracy.

### 5.3. Classification Accuracy for Various Window Sizes

**Figure 6:**
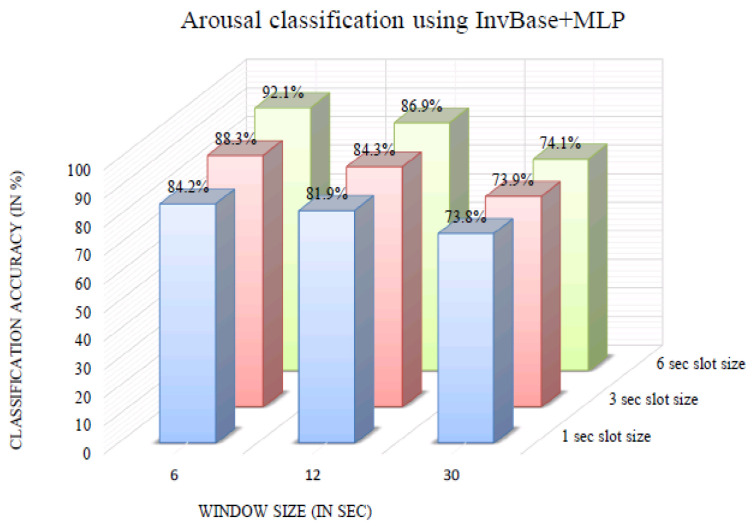
Arousal classification accuracy of the InvBase+MLP method for various window sizes.

**Figure 7:**
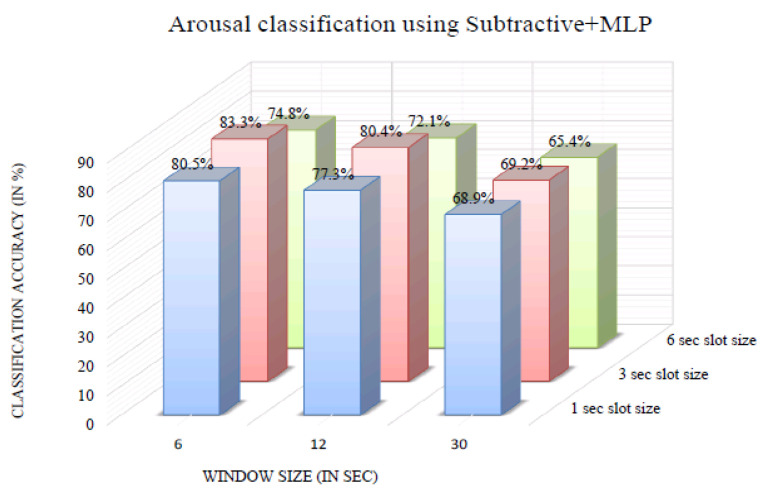
Arousal classification accuracy of the subtractive+MLP method for various window sizes.

**Figure 8:**
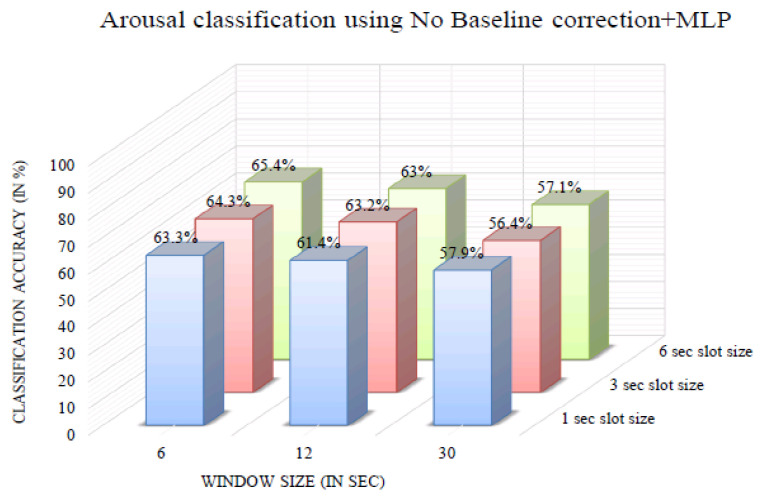
Arousal classification accuracy of NBC+MLP method for various window sizes.

The impact of varying the window size on classification accuracy was investigated. Figure 6 shows the arousal classification accuracy of the InvBase method+MLP for various window sizes. It was observed that in all the window sizes, the classification accuracy remained above 70% for the 1, 3, and 6 s slot sizes. Using the 6 s window produced a higher classification accuracy for 1 s, 3 s, and 6 s slot sizes than other window sizes. The arousal classification accuracy using the InvBase method+MLP was 92.1% for a 6 s slot size and 6 s window size, which was the highest. Figure 7 shows the arousal classification accuracy of the subtractive method+MLP with varying the window size. It was observed that for all the window sizes, the classification accuracy remained above 65% for the 1 s, 3 s, and 6 s slot sizes. Using the 6 s window size produced higher classification accuracy for 1 s, 3 s, and 6 s slot sizes than the other window sizes. The arousal classification accuracy using the subtractive method+MLP was 83.3% for the 3 s slot size and 6 s window size, which was lower than that of the InvBase+MLP method. Figure 8 shows the arousal classification accuracy of NBC+MLP with varying window size. We is observed that window size had a negative impact on arousal classification accuracy in the NBC+MLP method.

**Figure 9:**
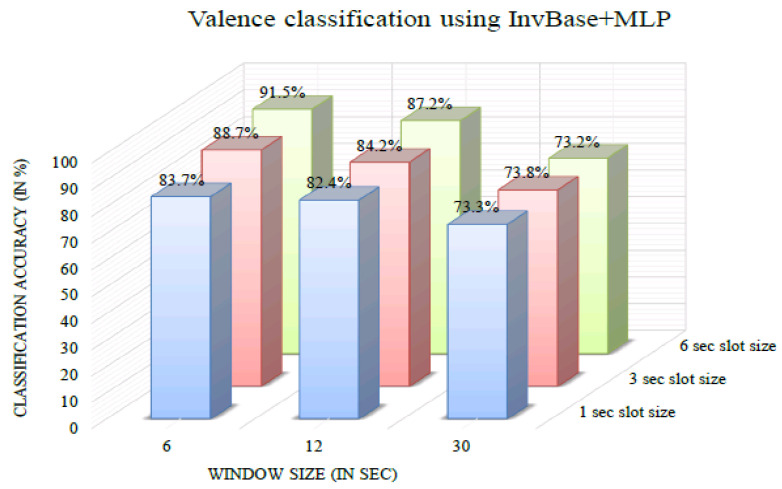
Valence classification accuracy of InvBase+MLP method for various window sizes.

**Figure 10:**
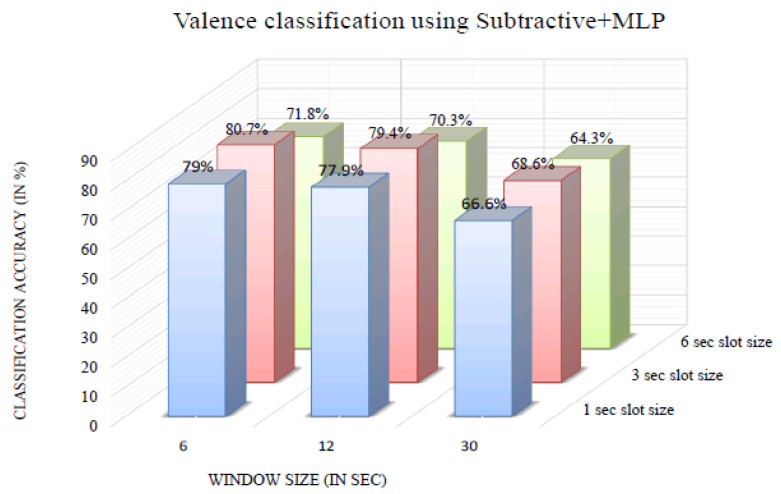
Valence classification accuracy of the subtractive+MLP method for various window sizes.

**Figure 11:**
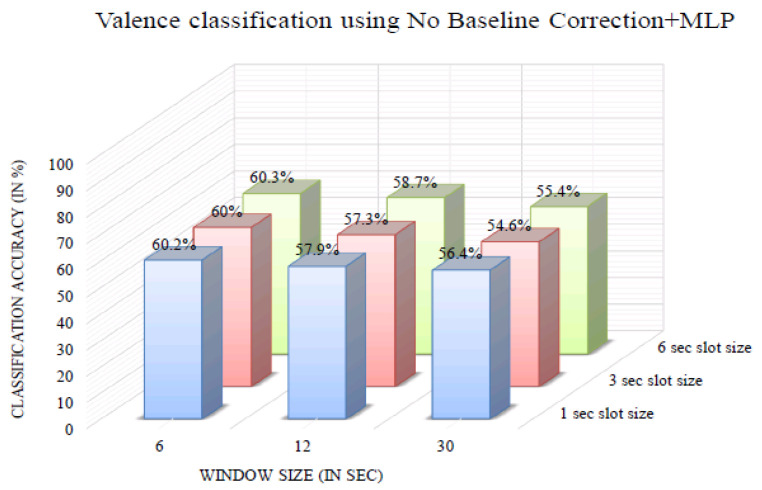
Valence classification accuracy of NBC+MLP method for variouswindow sizes.

Figure 9 shows the valence classification accuracy of the InvBase method+MLP for various window sizes. It was observed that for all the window sizes, the classification accuracy remained above 70% for 1 s, 3 s, and 6 s slot sizes. The 6 s window size produced higher classification accuracy for 1 s, 3 s, and 6 s slot sizes than the other window sizes. The valence classification accuracy was 91.5% for the 6 s slot size and 6 s window size. Figure 10 shows the valence classification accuracy of the subtractive method+MLP with varying window size. It was observed that for all the window sizes, the classification accuracy remained above 64% for the 1 s, 3 s, and 6 s slot sizes. Using the 6 s window size produced higher classification accuracy for 1 s, 3 s, and 6 s slot sizes than the other window sizes. The valence classification accuracy was 80.7% for the 3 s slot size and 6 s window size. Figure 11 shows the valence classification accuracy of the NBC+MLP method with varying window size. It was observed that window size had a negative impact on the valence classification accuracy in the NBC+MLP method.

### 5.4. Leave-One-Out Cross-Validation

**Figure 12:**
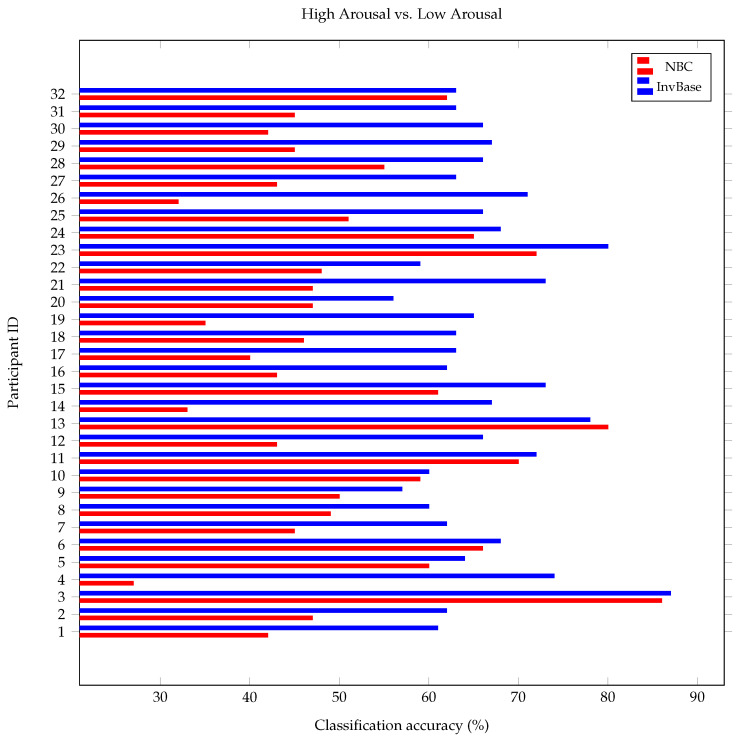
Comparison of arousal classification accuracy using InvBase method and NBC in leave one out cross validation.

**Figure 13:**
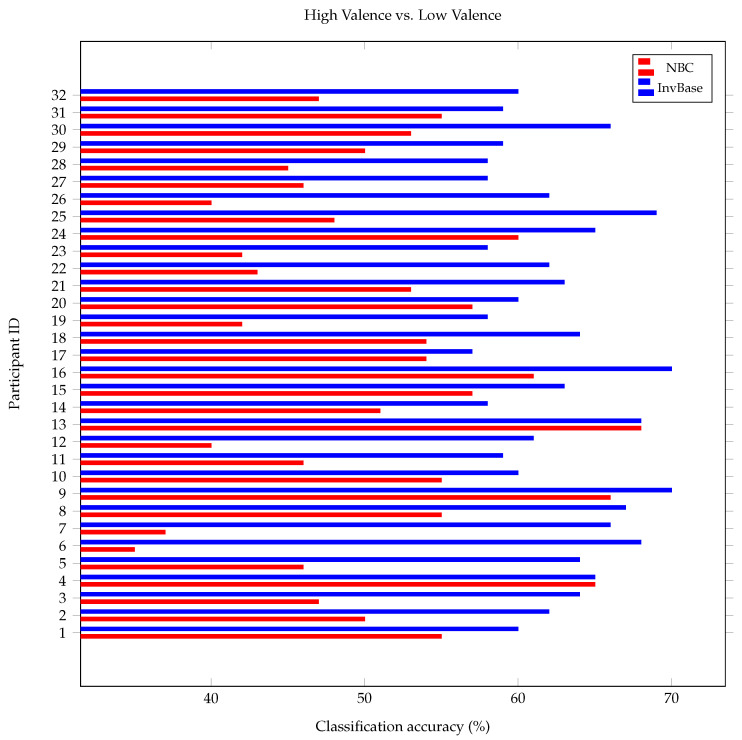
Comparison of valence classification accuracy using InvBase method and NBC in leave-one-out cross-validation.

In order to further evaluate the subject independence property of the InvBase method, leave-one-out cross-validation was performed on the DEAP dataset. The feature vectors extracted from one participant out of N subjects in the experiment were left out as the test set. The remaining feature vectors of N–1 participants were used to train the classifier. Furthermore, the MLP classifier was used, and slot and window sizes were set to 6 s, as the results in Section 5.3 indicated that the InvBase+MLP approach produced the highest classification accuracy with a 6 s slot and window size. The arousal classification accuracy obtained for each participant using the InvBase method and NBC approach is presented in Figure 12. The average arousal classification accuracies obtained by the classifier using the InvBase method and NBC were 66.40% and 51.13%, respectively. The valence classification accuracy obtained for each participant using the InvBase method and the NBC approach is presented in Figure 13. The average valence classification accuracies obtained by the classifier using the InvBase method and NBC were 62.59% and 50.72%, respectively. It was observed that the classification accuracy obtained on features extracted using the InvBase method was higher than that using the NBC approach for most of the participants. As the classifier was tested on an entirely different participant who was not presented during training, the improved classification accuracy validated the subject-independence property of the features extracted using the InvBase method.

### 5.5. Validation on Other Dataset

To verify the robustness and effectiveness of the proposed InvBase method, experiments were conducted on the SEED dataset in a similar fashion, where SEED stands for Shanghai Jiao Tong University (SJTU) emotion EEG dataset [37]. The SEED dataset consists of EEG recording of subjects obtained while they watched 15 different clips from Chinese films. Each video clip was used to elicit any one of the three types of emotion: positive, negative, or neutral. The EEG data of a subject contain recordings from 62 EEG channels, and they are categorized into three classes based on the type of emotional content. A multiclass classification problem was formulated in the context of the SEED dataset, and the observations were drawn, keeping the experimental setup similar to that used for the DEAP dataset.

The InvBase method showed a clear improvement in classification accuracies over the other methods. Similar to the results on the DEAP dataset, the MLP classifier provided superior classification accuracy on the SEED dataset. Using the MLP classifier, the highest improvement of 13.84% was recorded with a 1 s slot size and 12-window size for the InvBase method. Additionally, more than 8.5% improvement in classification accuracy using InvBase+MLP was observed with a 12 s window size for slot sizes of 1, 3, and 6 s. One important observation is that the 1 s slot size produced better classification on the SEED dataset, whereas, in the DEAP dataset, the highest classification accuracy was obtained for the 6 s slot size. The difference can be justified by the differences in the number of EEG channels and sampling frequency and by the different types of stimuli used. The EEG recordings of the SEED dataset have 62 channels compared with the 32 channels in the DEAP dataset. Additionally, the sampling frequency in the SEED dataset is 200 Hz, which is higher than that in the DEAP dataset. The higher sampling frequency and larger number of EEG channels may increase the resolution of emotion content in the EEG recording. Thus, in the SEED dataset, a higher classification accuracy using a smaller slot size was observed than in the DEAP dataset. For brevity, extensive experimental results are not included here.

### 5.6. Time Complexity Analysis

In this section, the time complexity analysis of the InvBase, subtractive, and NBC methods for baseline removal is discussed. In each baseline-removal method, three are three major steps: (1) FFT of the EEG signal, (2) point-wise removal operation, and (3) calculation of frequency band power. Let $t_{A}$ be the time required for calculating the FFT of an EEG signal with *n* sample points, $t_{B}$ be the time required for point-wise removal operation in a frequency spectrum having *f* frequency points, and $t_{C}$ be the time required for calculating the frequency band power. The total running time required for each baseline removal method in *c* EEG channels, $T_{R}$, is given in Equation (7).

(7) $$T_{R}=c*\left(T_{A}+T_{B}+T_{C}\right)$$

From Equation (7), the worst-case time complexity of the baseline-removal process can be represented as

$$O\left(T_{R}\right)=O\left(c*T_{A}+c*T_{B}c*T_{C}\right)$$

(8) $$O\left(T_{R}\right)=O\left(c*T_{A}\right)+O\left(c*T_{B}\right)+O\left(c*T_{C}\right))$$

As the worst-case time complexity of the FFT operation is $O(n*log_{2}\left(n\right))$, we can represent $O\left(c*T_{A}\right)=O\left(c*n*log_{2}\left(n\right)\right)$. Considering subtraction in the subtractive method and division in the InvBase method to be the unit operation in each frequency point, we can substitute $O(c*T_{B})$ wih O(c∗f). However, in the NBC method, $O(c*T_{B})=0$, as there is no point-wise baseline-removal operation. Lastly, the calculation of frequency-band power involves finding the mean power in five frequency bands over the frequency spectrum; therefore, we can substitute $O(c*T_{C})$ with O(c∗f). Substituting $O(c*T_{A})$, $O(c*T_{B})$, and $O(c*T_{C})$ in Equation (8), the worst-case time complexity for the InvBase, $T_{I}$, and subtractive, $T_{S}$, methods can be written as:

$$O\left(T_{Inv}\right)=O\left(T_{Sub}\right)=O\left(c*n*log_{2}\left(n\right)\right)+O\left(c*f\right)+O\left(c*f\right)$$

(9) $$O\left(T_{Inv}\right)=O\left(T_{Sub}\right)=O\left(c*n*log_{2}\left(n\right)\right)+O\left(c*f\right)$$

Similarly , substituting $O(c*T_{A})$, $O(c*T_{B})$, and $O(c*T_{C})$ in Equation (8), the worst-case time complexity for the NBC method can written as:

(10) $$O\left(T_{NBC}\right)=O\left(c*n*log_{2}\left(n\right)\right)+O\left(c*f\right)$$

From Equations (9) and (10), we concluded that all the three methods for baseline removal have a similar time complexity, i.e., $O\left(T_{Inv}\right)=O\left(T_{Sub}\right)=O\left(T_{NBC}\right)$.

## 6. Discussion

The results presented here indicate that the proposed baseline-removal technique provides significant improvement in classification accuracy compared with the subtractive and no-baseline-correction methods. Using multilayer perceptron, the classification accuracy improved by 29% over the no-baseline-correction method and 15% over the subtractive method. Experiments were conducted on various slot sizes, and we found that the 6 s slot size provided the highest classification accuracy and F1 score for both valence and arousal. Furthermore, varying the window size, classifications were performed, and the results indicated that increases in the window size result in decreases in classification accuracy. This is due to the fact that calculating the features over a large window size results in lower frequency resolution.

There are few studies that considered a baseline removal strategy. When compared with the existing studies, the proposed method outperforms other methods [8,18,19] in terms of classification accuracy. Another advantage of the proposed method compared with other existing methods is that it uses traditional machine learning models, which are relatively less complex. One of the limitations of this study is that we used fixed-size time windows while calculating the features. Furthermore, we did not use advanced machine learning techniques, such as CNN, for enhancing the classification accuracy. This study highlights the importance of baseline removal, as the accuracy of the classifier directly depends on the quality of the input data. The results showed that the InvBase method of baseline elimination outperforms existing state-of-the-art baseline-removal methods in EEG-based emotion recognition systems. The ability of the proposed method to remove baseline noise from EEG signals provides room for progress in EEG-based emotion detection. Many researchers have reported improved classification accuracy using deep learning techniques [38,39]. The InvBase method with deep learning is a promising option for further improving the classification accuracy.

## 7. Conclusions

In this study, a novel method called InvBase was developed for baseline removal, and features were extracted for the classification of emotion. This method of baseline elimination resulted in higher classification accuracy for valence and arousal classifications in comparison with the subtractive method. The results suggested that the baseline removal method increases the classification accuracy with very simplistic features such as the mean and variance of EEG band powers. In comparison with the no-baseline-correction method, the improvement in average classification accuracy obtained by using the InvBase method established that the features extracted from baseline-removed EEG signals suppress the variation introduced by the subject’s baseline, thus resulting in close to subject-independent features. Because the baseline power present in EEG signals introduces noise to the extracted features, eliminating the baseline power using the InvBase method results in a significant improvement in emotion classification accuracy. Furthermore, an important conclusion in this study is that emotion features were dominant in time slots ranging from 1 s to 12 s. Additionally, we observed that the InvBase+MLP approach gave the highest classification accuracy and F1 score with a 6 s slot size and window size. Fewer features need to be extracted from EEG data with the InvBase method and subtractive method compared with other methods. Thus, training a classifier requires less computation time because the dimensionality of the features is decreased.

## Figures and Tables

**Table 1:** Sample size of different valence and arousal binary classes (total number of samples = 1280).

Class	No. of Samples
High Valence	587
Low Valence	693
High Arousal	620
Low Arousal	660

**Table 2:** Arousal classification accuracies and F1 scores of InvBase, subtractive, and NBC methods using MLP for various slot sizes.

Slot Size	InvBase Method		Subtractive Method		NBC Method	
**(s)**	**Acc (%)**	**F1**	**Acc (%)**	**F1**	**Acc (%)**	**F1**
1	81.9	0.816	77.2	0.754	61.42	0.591
3	84.3	0.836	80.4	0.798	63.2	0.614
6	86.9	0.865	72.1	0.713	63.0	0.619
12	85.1	0.848	68.2	0.668	62.6	0.604
15	76.6	0.763	67.4	0.666	63.2	0.610
30	68.4	0.675	61.1	0.603	57.6	0.542

**Table 3:** Arousal classification accuracies and F1 scores of InvBase, subtractive, and NBC methods using SVM for various slot sizes.

Slot Size	InvBase Method		Subtractive Method		NBC Method	
**(s)**	**Acc (%)**	**F1**	**Acc (%)**	**F1**	**Acc (%)**	**F1**
1	79.7	0.790	68.4	0.628	63.6	0.592
3	83.1	0.820	70	0.653	62.6	0.579
6	86.0	0.854	66.4	0.642	62.9	0.582
12	84.0	0.835	64.3	0.612	63.2	0.587
15	72.4	0.707	63.3	0.586	63	0.581
30	64.0	0.611	61.9	0.544	61.3	0.556

**Table 4:** Arousal classification accuracies and F1 scores of InvBase, subtractive, and NBC methods using kNN for various slot sizes.

Slot Size	InvBase Method		Subtractive Method		NBC Method	
**(s)**	**Acc (%)**	**F1**	**Acc (%)**	**F1**	**Acc (%)**	**F1**
1	74.4	0.694	71.2	0.687	63.7	0.579
3	78.2	0.755	72.1	0.695	63.6	0.595
6	76.9	0.751	67.8	0.645	63.5	0.594
12	76.4	0.747	65.4	0.620	63.7	0.597
15	69.7	0.646	64.2	0.600	63.3	0.591
30	58.3	0.477	61.4	0.564	60.5	0.552

**Table 5:** Valence classification accuracies and F1 scores of InvBase, subtractive, and NBC methods using MLP with various slot sizes.

Slot Size	InvBase Method		Subtractive Method		NBC Method	
**(s)**	**Acc (%)**	**F1**	**Acc (%)**	**F1**	**Acc (%)**	**F1**
1	82.4	0.807	77.9	0.755	57.9	0.505
3	84.2	0.831	79.4	0.773	57.3	0.508
6	87.2	0.861	70.3	0.673	58.7	0.544
12	85.8	0.845	65.3	0.611	58.6	0.539
15	74.9	0.727	64.2	0.599	57.4	0.544
30	67.5	0.675	59.6	0.542	55.2	0.498

**Table 6:** Valence classification accuracies and F1 scores of InvBase, subtractive, and NBC methods using SVM with various slot sizes.

Slot Size	InvBase Method		Subtractive Method		NBC Method	
**(s)**	**Acc (%)**	**F1**	**Acc (%)**	**F1**	**Acc (%)**	**F1**
1	79.9	0.766	67	0.555	58.6	0.432
3	82.2	0.798	68.4	0.590	58.4	0.426
6	84.5	0.821	62.2	0.492	57.8	0.418
12	82.5	0.794	60.3	0.455	57	0.413
15	71.0	0.659	58.8	0.453	57.6	0.411
30	62.3	0.545	55.6	0.384	56.3	0.406

**Table 7:** Valence classification accuracies and F1 scores of InvBase, subtractive, and NBC methods using kNN with various slot sizes.

Slot Size	InvBase Method		Subtractive Method		NBC Method	
**(s)**	**Acc (%)**	**F1**	**Acc (%)**	**F1**	**Acc (%)**	**F1**
1	73.9	0.649	69.5	0.629	59.1	0.485
3	77	0.728	70.4	0.636	58.5	0.479
6	75.4	0.696	64.7	0.562	59.2	0.489
12	74.7	0.695	61.6	0.516	58.2	0.478
15	69.0	0.611	60.6	0.503	57.4	0.460
30	59.3	0.431	57.8	0.468	54.4	0.429

## Data Availability

Not applicable.

## Abbreviations

The following abbreviations are used in this paper:

ANS	autonomous nervous system
AI	asymmetry Index
ASM	asymmetry measure
CNN	convolutional neural network
CSP	common spatial patterns
DE	differential entropy
DEAP	database for emotion analysis using physiological signals
EEG	electroencephalogram
EOG	electrooculogram
GSR	galvanic skin response
HA	high-arousal
HMI	human–machine interaction
HOC	higher-order crossings
HV	high-valence
kNN	k-nearest neighbor
LA	low-arousal
LV	low-valence
MLP	multilayer perceptron
NBC	no baseline correction
PSD	power spectral density
RNN	recurrent neural network
SEED	SJTU emotion EEG dataset
SFV	spatial feature vector
SVM	support vector machine
TFV	temporal feature vector
WT	wavelet transform
